# Development and validation of the digital competence framework and scale for pre-service physical education teachers

**DOI:** 10.1038/s41598-026-63030-z

**Published:** 2026-07-23

**Authors:** Lei Li, Zhenhui Pan, Yaqin Ren, Cheng Xiao

**Affiliations:** School of Physical Education, Guangzhou Sport University, No. 1268, Guangzhou Avenue Middle, Tianhe District, Guangzhou, 510500 Guangdong Province China

**Keywords:** Pre-service physical education teachers, Digital competence, Subject-specific framework, Exploratory sequential mixed-methods research, Education, Psychology, Psychology

## Abstract

General teacher Digital Competence (DC) frameworks, which lack a subject-specific pedagogical perspective, are limited in their ability to effectively guide technology integration practices in physical education contexts. Using an exploratory sequential mixed-methods design, this study developed and validated a subject-specific framework and measurement instrument for the DC of Pre-service Physical Education Teachers (PSPETs). The results showed that the PSPETs’ DC framework comprises Digital Resources for Physical Education Teaching, Integration and Implementation in Physical Education Teaching, Assessment and Feedback in Physical Education Teaching, Digital Ethics and Safety in Physical Education Teaching, Professional Activities, Fostering Students’ Use of Digital Technology for Health Maintenance, and Personality Traits in Digital Technology Application. Based on this framework, a measurement instrument was developed and evaluated through item analysis and cross-validation using 1,437 valid questionnaires. The results confirmed the instrument’s satisfactory reliability and validity, as well as the good fit of its seven-factor structural model. Further survey results based on 3,128 PSPETs indicated that institutional tier, GPA Ranking, academic year, and city tier were positively associated with DC, whereas first-generation college student status and low-income family status were negatively associated with DC. By incorporating a subject-specific perspective, this study contributes a domain-specific framework that integrates technology integration with subject-based educational objectives. It also provides a reference for the design of Physical Education Teacher Education (PETE) curricula, the diagnosis of DC, and differentiated professional development.

**Supplementary Information:**

The online version contains supplementary material available at 10.1038/s41598-026-63030-z.

## Introduction

Over the past several decades, the digital transformation of education has remained a central global priority. The widespread adoption of digital tools and technologies has generated continually evolving demands for teachers’ knowledge, skills, and critical thinking, while also requiring them to be adequately prepared for the broad range of competencies needed in present and future classrooms^1^(Yang, 2024).

However, a substantial body of research has demonstrated a significant gap between the digital competence (DC) of both pre-service and in-service teachers and the demands of actual teaching practice^2,3^. This gap can be traced to the lack of digital practices during teacher education and the inadequacy of corresponding curricular content within teacher preparation systems^4^. In response to this challenge, organizations such as the United Nations Educational, Scientific and Cultural Organization (UNESCO) and the European Commission (EC) have successively developed influential generic frameworks for teacher digital competence (TDC). These frameworks provide important foundations for defining, guiding, and assessing teachers’ digital competence^5,6^. Many scholars have also drawn on these generic frameworks to assess the digital competence of pre-service teachers across different subject areas^7–9^.

As research has progressed, scholars have increasingly recognized the limitations of generic frameworks from the perspective of subject-specific pedagogy. The requirements for digital competence differ fundamentally across teaching contexts and disciplines. This recognition has prompted the development of subject-specific digital competence frameworks in areas such as science, reading, and foreign-language education, with the aim of providing more effective guidance for technology integration in subject teaching^10–12^. Physical education involves outdoor activities as well as processes such as motor-skill demonstration and workload monitoring, requiring the use of technologies such as sports watches, heart-rate monitoring straps, and video-analysis tools to support instruction and assessment^13^. Existing digital competence frameworks remain insufficiently sensitive to the complex relationship between pedagogy and technology in physical education^14^, making the need for a specialized digital competence framework particularly pressing in this field. Nevertheless, current research has largely remained at the level of simply applying generic frameworks. Consequently, the development of digital competence among pre-service physical education teachers (PSPETs) lacks specific objectives and effective measurement instruments.

Accordingly, this study addresses three research questions: (1) What is the conceptualization of digital competence among PSPETs, and what dimensions should its framework contain? (2) How can a measurement instrument based on a digital competence framework for PSPETs be developed and validated? and (3) Is the digital competence of PSPETs influenced by their individual educational backgrounds and family-capital conditions? This study adopts an exploratory sequential mixed methods design and proceeds through three stages: qualitative interviews, scale validation, and questionnaire-based investigation.

## Literature review

### Digital literacy and digital competence

As digital technologies have become increasingly prevalent in education, the terminology used in this area has expanded considerably, yet substantial definitional debates have also emerged. In particular, “digital literacy” and “digital competence” are often used interchangeably^1^. However, the two concepts carry different meanings and have distinct practical implications for how Physical Education Teacher Education (PETE) programmes define and assess the capabilities that future teachers need to enact in practice.

The concept of digital literacy can be traced to the pioneering work of Gilster (1997), who initially defined it as an individual’s basic ability to access, integrate, and understand information in a digital information environment^15,16^. UNESCO subsequently expanded the concept to encompass the retrieval, evaluation, creation, and communication of information^17^. Despite this expansion, the term “digital literacy” has continued to emphasize the development of operational skills and information-processing abilities in many policy documents and teacher-education contexts, often without adequately incorporating pedagogical judgement and contextual adaptability^18^.

By contrast, “digital competence” has emerged as a more inclusive concept that better captures the complex demands faced by professionals in digital environments. It refers to the ability to use digital technologies confidently, critically, and responsibly across diverse professional contexts^5,6^. Falloon (2020) argued that skills-centred digital literacy frameworks are insufficient to prepare teachers for the complexity of contemporary classrooms and accordingly proposed an expanded concept of Teacher Digital Competence^19^. From a functionalist perspective, teachers’ digital competence is more appropriately understood as a form of dynamic agency that enables teachers to make pedagogical decisions regarding the deployment of technology in pursuit of specific subject-related goals^20^. This perspective indicates that effective technology use requires considerably more than operational proficiency. Teachers must also be able to adapt digital tools to particular disciplines, student groups, and instructional contexts.

In physical education, although digital competence related to instructional design and implementation shares substantial commonalities with that of other subjects, the distinctive nature of the field has also generated a range of specialized competencies closely associated with sport instruction and physical activity. For example, wearable devices such as heart-rate monitoring straps, fitness trackers, and accelerometers can be used to collect students’ exercise-load and physiological data, thereby supporting real-time feedback and personalized instruction^13,21–23^. Video-analysis tools are also being increasingly applied to motor-skill instruction. Through slow-motion replay, postural comparison, and movement decomposition, teachers can help students understand and correct technical movements more precisely^24^. Furthermore, with the rapid development of artificial intelligence, emerging technologies such as AI-assisted movement assessment and virtual-reality scenario simulation have begun to enter physical education practice^25,26^, further expanding the potential scope of technology integration.

### Generic and subject-specific frameworks

The past decade has witnessed a proliferation of generic frameworks for teacher digital competence. Representative examples include the International Society for Technology in Education (ISTE) Standards for Educators^27^, the UNESCO ICT Competency Framework for Teachers (ICT-CFT)^28^, the European Commission’s Digital Competence Framework for Educators (DigCompEdu)^29^, and China’s Standards for Teachers’ Digital Literacy^30^.

More specifically, the ISTE Standards for Educators conceptualize teachers as occupying seven roles, including learner, leader, and designer, and emphasize the cultivation of innovative educational cultures and digital citizenship^27^. The UNESCO ICT-CFT organizes competencies into three progressive levels—knowledge acquisition, knowledge deepening, and knowledge creation—thereby providing a developmental trajectory for teacher competence^28^. The European Commission’s DigCompEdu outlines six areas of educators’ professional practice: professional engagement, digital resources, teaching and learning, assessment, empowering learners, and facilitating learners’ digital competence. It therefore provides a comprehensive guide for professional development^29^. China’s Standards for Teachers’ Digital Literacy likewise offers a strongly practice-oriented framework, with its dimensions closely aligned with instructional processes such as design, implementation, and assessment^30^.

Despite differences in structure and emphasis, these frameworks share several conceptual commitments: a student-centred orientation; the assumption that technology should be integrated to enhance rather than replace teaching practice; an emphasis on continuous professional learning; and attention to ethical responsibilities in digital environments. These common commitments make generic frameworks valuable references for educational policy and teacher-education programme design across national contexts. Nevertheless, the common requirements that generic frameworks establish for teachers across all subjects are achieved largely at the expense of subject specificity^20^. As noted in previous research, generic frameworks provide little specific guidance on how technology integration should be adapted to the distinctive cognitive practices, pedagogical forms, and learning objectives of particular subjects^31^.

Recognition of these limitations has prompted the development of digital competence frameworks in several subject areas. In science education, the DiKoLAN framework distinguishes between subject-specific competence domains and cross-disciplinary generic competence domains, acknowledging that science teachers require specialized competencies related to laboratory environments, data collection, and scientific modelling^11^. In English-language teaching, Moorhouse et al. (2024) identified five dimensions of digital competence among beginning English teachers in Hong Kong, including technological proficiency, pedagogical compatibility, preparing students for the digital world, and risk and ethical awareness. These dimensions reflect the distinctive communicative and intercultural demands of language teaching^12^.

In physical education, previous studies have identified a structure of physical education teachers’ digital competence encompassing digital awareness, digital knowledge and skills, digital application, digital social responsibility, and professional development^32,33^. Rahman et al. (2026) and Ahmad Kamil et al. (2025) have also investigated the assessment and training of physical education teachers’ digital competence based on the DigComp framework^34,35^. However, these frameworks have generally failed to pay sufficient attention to the complex relationship between pedagogy and technology in physical education. Their dimensional structures are almost identical to those of generic frameworks and have not been naturally derived from the practical logic of physical education as a discipline.

## Methods

This study adopted an Exploratory Sequential Mixed Methods (ESMM) design^36^. The study was conducted in three sequential phases. In Phase 1, Grounded Theory (GT) was used to explore and construct a theoretical framework and identify the core dimensions of digital competence among Pre-service Physical Education Teachers (PSPETs). In Phase 2, the Survey Scale of Digital Competence among PSPETs was developed based on the theoretical framework, which was then empirically validated. In Phase 3, the validated scale was administered in a large-scale survey to examine differences in PSPETs’ digital competence across multiple demographic variables. The study was approved by the Ethics Committee of Guangzhou Sport University (approval no. 2026LCLL-005).

### Research instruments

First, one-to-one semi-structured interview data were collected. The core components of the interview protocol were designed using the STAR (Situation, Task, Action, Result) behavioural-event interview method^37,38^, covering four modules: background information, recollection of critical incidents, in-depth analysis of the incidents, and reflection and abstraction of competencies. During the iterative process of data collection and preliminary analysis, participants were selected in accordance with the principles of theoretical sampling, and the interview protocol was dynamically revised based on concepts that emerged from the preliminary analysis. All interviews were conducted by the first author. Sixteen interviews were conducted through online video conferencing, whereas nine were conducted face-to-face. Each interview lasted approximately 30–60 min and was audio-recorded in its entirety after informed consent had been obtained. In addition to the interviews, supplementary materials, including lesson plans and reflective journals, were collected to corroborate the interview data. All interview recordings were transcribed verbatim, resulting in approximately 59,000 Chinese characters of textual data. Together with the supplementary materials, these data constituted the database for the qualitative analysis.

Second, a researcher-developed Questionnaire on Digital Competence among PSPETs was used. The questionnaire comprised two sections: background information and a core scale. The background section included seven sociodemographic and educational variables, such as gender, first-generation college student status, and family economic circumstances. The initial item pool for the core scale was generated from the interview data and relevant literature. Forty-eight items were developed across seven dimensions and scored on a five-point Likert scale.

To establish content validity, four professors independently reviewed the initial items^39^. Two reviewers were Physical Education Teacher Education (PETE) faculty members whose research focused on digital teaching, and two were secondary-school physical education teachers with experience in digital teaching. The review form provided the operational definition of each dimension and the items assigned to it. Items were rated on a four-point scale, ranging from 1 (not relevant) to 4 (highly relevant). An item-level Content Validity Index (I-CVI) of ≥ 0.78 was used as the retention criterion^40^. Six items with an I-CVI below 0.78 were deleted. On the basis of the experts’ qualitative recommendations, four semantically overlapping items and four items that deviated from the operational definitions of their dimensions were subsequently removed. In addition, one item was reworded as a reverse-scored item to reduce response-set bias. After these revisions, 34 items remained for pilot testing.

The average scale-level Content Validity Index (S-CVI/Ave), recalculated using the retained items, was 0.92. In addition, the inter-rater reliability results based on Kendall’s Coefficient of Concordance (Kendall’s W) indicated good agreement among the expert raters. Subsequently, 253 PSPETs who met the inclusion criteria were recruited for the pilot test. Common Method Bias (CMB) was controlled through anonymous responses, randomization of item order, inclusion of reverse-scored items, and embedded attention-check items.

Item analysis was first conducted using the extreme-groups method. Participants were divided into high- and low-score groups, each representing 27% of the total sample, and independent-samples t tests were conducted^41^. Using *p* < .05 as the significance threshold, one item that did not reach statistical significance was deleted. The Corrected Item-Total Correlation (CITC) was then calculated, and one item with a CITC below 0.30 was removed^42^.

To further examine the structural validity of the items, an Exploratory Factor Analysis (EFA) was conducted on the remaining 32 items using Principal Component Analysis (PCA) with Promax Oblique Rotation. The number of factors was determined based on eigenvalues greater than 1 and inspection of the scree plot. All item factor loadings exceeded 0.40, and no substantial cross-loadings were observed. The factor structure was therefore considered clear, and all 32 items were retained for the formal survey. The complete scale is provided in Supplementary Material 1.

Third, the Questionnaire on Digital Competence among PSPETs was used as the final measurement instrument. The total scale demonstrated good internal consistency, with Cronbach’s alpha of α = 0.939. The EFA and Confirmatory Factor Analysis (CFA) jointly supported the hypothesized seven-factor structure. On the basis of relevant statistical literature, conventional fit criteria were used to evaluate model fit^43,44^. The final model showed a good fit to the data: χ²/df = 2.08, Comparative Fit Index (CFI) = 0.973, Tucker–Lewis Index (TLI) = 0.969, and Root Mean Square Error of Approximation (RMSEA) = 0.039. The Average Variance Extracted (AVE) ranged from 0.67 to 0.72, and Composite Reliability (CR) ranged from 0.86 to 0.92. These results indicated that the scale was a stable, valid, and structurally well-defined instrument for subsequent large-scale investigations and group-difference analyses.

### Sample size and data-collection procedures

In Phase 1, Maximum Variation Purposive Sampling was combined with theoretical sampling. Participants were recruited through the researchers’ social networks and online channels. To ensure that the sample reflected both theoretical and practical perspectives and captured the application of digital technologies in authentic teaching contexts, participants were required to meet all of the following criteria: (1) they had completed at least one compulsory or elective course related to educational technology; (2) they had experience with microteaching or a short-term teaching practicum; and (3) they were currently enrolled as undergraduate or master’s students majoring in physical education.

During the initial stage, 15 PSPETs from different regions, institutions, genders, and educational levels were recruited through purposive sampling. As the data analysis progressed, the research team shifted to theoretical sampling^45^. During this stage, five students from the researchers’ institution, four university faculty members responsible for PETE courses, and two in-service primary- or secondary-school physical education teachers with experience in digital teaching were recruited to provide perspectives from different professional groups. The final qualitative sample comprised 26 participants: 20 PSPETs, four university PETE educators, and two primary- or secondary-school physical education teachers. All PSPET participants had completed at least one educational-technology course and had experience with microteaching or a short-term teaching practicum.

In Phase 2, the revised questionnaire was distributed online through the National School Physical Education Alliance project. Because the primary purpose of this phase was to validate the theoretical model and measurement scale, student participants were required to meet the same recruitment criteria as those used in Phase 1. A stratified cluster sampling design was adopted. The sampling frame was stratified by geographical region and institutional level. Three universities were then selected from each stratum as the primary sampling units. Within the selected universities, intact classes in physical education programmes served as the cluster sampling units. The researchers contacted student coordinators through online communities, and the coordinators distributed the questionnaire within their respective class groups.

A total of 1,872 questionnaires were returned. Data were screened according to predefined criteria. Invalid responses were removed if they had an excessively short completion time, failed the embedded attention-check item, or displayed an identical response pattern across all items. A total of 1,437 valid responses remained, yielding a valid response rate of 76.8%. The total sample was then randomly divided into two subsamples: Subsample A (*n* = 719) was used for item analysis and EFA, whereas Subsample B (*n* = 718) was used for CFA and reliability testing.

For Subsample A, the ratio of sample size to number of items substantially exceeded the commonly recommended ratio of 10:1, ensuring the stability of the results^46^. For Subsample B, the sample size exceeded 500, meeting the “excellent” criterion for CFA sample size and ensuring stable estimation of the model parameters^47^.

In Phase 3, the validated scale was administered through another stratified cluster sampling procedure. A large-scale online survey was conducted among physical education students at 25 sport universities, normal universities, and comprehensive universities located in eastern, southern, and northern China. To examine differences in PSPETs’ digital competence across academic years, participants were required to be currently enrolled undergraduate or master’s students majoring in physical education.

A total of 3,650 questionnaires were returned. After applying the same data-screening criteria used in Phase 2, 3,128 valid responses remained, corresponding to a valid response rate of 85.7%. According to Green’s (1991) criterion, *N* > 50 + 8k, where k represents the number of predictors, the minimum required sample size was 146^48^. The actual sample size (*N* = 3,128) therefore provided sufficient statistical power for the planned analyses. The demographic characteristics of the samples in Phases 2 and 3 are presented in Supplementary Material 1.

### Data-analysis methods

First, the procedural GT approach was followed, and the qualitative data were analysed through three levels of coding. Constant comparison across interview transcripts was conducted throughout the analysis to identify similarities and differences among concepts and categories. Memos were also written to document analytic reflections, preliminary assumptions, and the evolving relationships among categories^45^. NVivo 14 was used throughout the process to assist with data management.

During open coding, the interview transcripts were analysed line by line. Original statements were divided into the smallest meaningful units and assigned conceptual labels. Wherever possible, participants’ own words and gerund phrases were used to remain closely grounded in the data. Through constant comparison, overlapping or conceptually inclusive concepts were merged, concepts with weak associations with the central themes were removed, and the remaining concepts were grouped into subcategories according to their shared core meanings. The initially developed subcategories were then reviewed a second time. Categories with unclear boundaries or similar meanings were merged, and the final structure of the subcategories was established^45,49^.

During axial coding, the subcategories were compared with one another to identify similarities and differences in their functional orientations. Subcategories with internal conceptual coherence were grouped into higher-order main categories^45,50^. During selective coding, the relationships among the categories were examined through constant comparison and memo writing. The core category was identified, and the main categories were integrated around it by constructing a paradigm model and developing a storyline^45^.

To assess coding reliability, two doctoral candidates specializing in physical education were invited to serve as independent coders. The transcripts were stratified by participant type, and 30% of the transcripts from each stratum were randomly selected for independent coding^51^. Inter-coder reliability was assessed using Cohen’s Kappa Coefficient. The resulting Cohen’s κ was 0.81, indicating substantial agreement^52^. Coding disagreements were resolved through team discussion. After the coding scheme had been revised accordingly, the first author completed the remaining coding^49^.

Sampling and data analysis proceeded concurrently until no new concepts or categories emerged from additional transcripts^45^. This point was reached during the analysis of the 21st transcript, after which two consecutive transcripts produced no new codes or categories. To confirm theoretical saturation, four remaining interview transcripts, representing approximately 15% of the total sample, were subjected to confirmatory analysis by a team member who had not participated in the initial coding^50^. No new categories or properties emerged.

To address researcher reflexivity, the first author maintained a reflexive journal throughout the study. The journal documented pre-existing assumptions regarding PSPETs’ digital competence, potential analytic biases arising from the researcher’s own experiences, and ongoing reflections on the data-collection and coding processes^50^. The journal was periodically reviewed by the research team. Team members who had not participated in the coding, together with the corresponding author, critically examined the coding logic and preliminary interpretations to help control researcher bias. Member checking was conducted after the preliminary framework had been developed to establish the credibility of the findings^52,53^. Participants who had taken part in the interviews received the complete theoretical framework and an explanation of the coding procedures by email. They were invited to provide written feedback on the accuracy of the categories, the consistency of the relationships among categories, and any potential omissions.

Second, item analysis was conducted on the total sample using the critical-ratio method and item-total correlation analysis. Cronbach’s alpha was calculated to assess the internal consistency of the scale. PCA was used for factor extraction, and Promax Oblique Rotation was applied for factor rotation^54^. EFA was conducted using Subsample A (*N* = 719). The number of factors was determined through parallel analysis and the criterion of eigenvalues greater than 1. CFA was subsequently conducted using AMOS 26.0 with Subsample B (*N* = 718).

Third, multiple linear regression analysis was conducted to examine the net effects of different background variables on PSPETs’ digital competence. Before the main analysis, CMB was assessed using Harman’s Single-Factor Test. An unrotated PCA was performed on all items, with a first-factor contribution of less than 50% of the total variance used as the threshold^55^. The results indicated that CMB did not pose a serious threat.

In addition, Multi-Group Confirmatory Factor Analysis (MG-CFA) was used to examine measurement invariance across seven groups of background variables, including gender and academic stage. Configural invariance, metric invariance, and scalar invariance were tested sequentially. Measurement invariance was considered supported when ΔCFI ≥ − 0.01 and ΔRMSEA ≤ 0.015^56^.

The total digital competence score was specified as the dependent variable. Seven background variables were entered as independent variables: gender, first-generation college student status, low-income family status, institutional level, academic year, GPA ranking, and the tier of the city in which the institution was located. The dummy coding of categorical variables is provided in Supplementary Material 1.

Parameters were estimated using Ordinary Least Squares (OLS). Differences between groups were evaluated through tests of the significance of regression coefficients. Standardized and unstandardized coefficients were used to assess the relative explanatory power and practical effect sizes of the predictors, respectively. Overall model significance and explanatory power were evaluated using the F test and adjusted R². Model diagnostics included the Variance Inflation Factor (VIF) and the Durbin–Watson statistic^57^.

Previous research has suggested that institutional resources and teaching quality may partially moderate the effect of family socioeconomic status on academic achievement^58^. Accordingly, two sets of interaction terms—low-income family × institutional level and first-generation college student status × institutional level—were entered sequentially after the main-effects model. Model comparisons and F tests of ΔR² were used to evaluate the incremental explanatory power of the interaction terms and to examine whether institutional level moderated the influence of family capital on digital competence in the Chinese context.

## Results

### Results of the three-level coding of the digital competence framework for PSPETs

This study followed the procedural GT approach and systematically analysed the interview data through three levels of coding. Open coding yielded 84 initial concepts (see Supplementary Material 1) and 25 subcategories. Through axial coding, these subcategories were further grouped into seven main categories. A total of 362 valid references were generated throughout the analysis. Table 1 and Supplementary Material 1 present the coding process from the raw data to the main categories, together with representative quotations and their corresponding initial concepts.

During the early stage of open coding, the researchers noted that several participants raised issues of equity, although they expressed these concerns in different ways. P06 described a personal experience in which some students were excluded during a practicum because of insufficient equipment, whereas P10 explicitly stated that digital competence should include considerations of equity. Initially, these two types of statements were coded as separate initial concepts. However, constant comparison indicated that they shared a core meaning: unequal participation caused by differences in technological conditions. They were therefore merged into “recognizing participation disparities caused by technological conditions” (OC-049), which was assigned to D1. After several rounds of comparison and refinement, four subcategories (D1–D4) were established, each representing a different type of boundary associated with technology use. During selective coding, the researchers further examined which subcategories could be integrated into a higher-order main category. The comparison showed that D1–D4 all referred to the constraints and behavioural boundaries that teachers should observe.

**Table 1:** Example of the three-level coding process.

Original quotation	Initial concept	Subcategory	Main category
“Is technology being used to make the class better, or merely to make the class look more advanced? If the technology itself is creating new forms of inequality, what is the point of using it?” (P06—03, PST)	OC-049 Focusing on participation inequalities caused by differences in technological conditions	D1 Ensuring educational equity in digital environments	D Digital ethics and safety in Physical Education Teaching
“You have to recognize that not everyone has access to the same equipment. You cannot prevent a student from participating in physical education simply because they do not have a smartwatch.” (P10—04, PST)	OC-049 Focusing on participation inequalities caused by differences in technological conditions		
“During my practicum, I assigned an extracurricular exercise-tracking task that required students to use a mobile-phone app. Some students said that they did not have smartphones. That was when I realized that I had actually excluded some students.” (P12—08, PST)	OC-049 Focusing on participation inequalities caused by differences in technological conditions		
…			
“The data generated in physical education are different from academic report-card data. Physical-fitness data include students’ physical characteristics, athletic abilities, and even health conditions. If such data are leaked, the harm could be much greater than that caused by the disclosure of academic scores.” (P19—09, PETE)	OC-052 Preventing the leakage of students’ health data	D2 Observing data ethics and protecting privacy	
…			
“When using images or videos from the internet, their sources should be listed at the end of the teaching materials. I did not pay attention to this in the past, but later someone pointed out that it was a copyright issue.” (P12—06, IST)	OC-055 Citing sources when using online materials	D3 Observing digital copyright regulations	
…			
“Wearable devices essentially extend the teacher’s senses by allowing the teacher to see things that cannot be detected with the naked eye. However, they cannot make decisions on behalf of the teacher. The teacher still has to make the judgement, because data cannot be trusted completely.” (P07—11, PST)	OC-058 Using wearable devices to identify exercise-related risks	D4 Warning of and monitoring students’ exercise-related risks	
…			

The identifiers in the quotation table include the participant number (e.g., P06) and the sequence number of the original statement (e.g., 03), allowing the source of each quotation to be traced.

PST = Pre-service Teacher; PETE = Physical Education Teacher Educator; IST = In-service Teacher. OC-XXX denotes an initial concept.

The integration process also involved discussion regarding whether “observing digital copyright regulations” (D3) and “warning of and monitoring students’ exercise-related risks” (D4) overlapped conceptually. Following team discussion, the researchers concluded that D4 concerned the boundaries governing the collection and use of student information, whereas D3 concerned the rules governing the use of others’ intellectual products. Because the two subcategories involved different objects and derived from different sources of regulation, they were retained as independent categories. D1–D4 were ultimately integrated into the main category “digital ethics and safety in Physical Education Teaching” (D). The complete coding structure and distribution of references across categories are presented in Table 2.

**Table 2:** Three-level coding results for the digital competence framework for PSPETs.

No.	Main category	Reference frequency	No.	Subcategory	Reference frequency
A	Digital resources for physical education teaching	92	A1	Searching for physical education teaching resources	38
			A2	Identifying physical education teaching resources	18
			A3	Managing physical education teaching resources	16
			A4	Producing physical education teaching resources	20
B	Integration and implementation of physical education teaching	88	B1	Analysing learners’ characteristics based on evidence	15
			B2	Designing physical education teaching activities	32
			B3	Implementing physical education teaching plans	28
			B4	Managing the class with the support of digital technologies	13
C	Assessment and feedback in physical education teaching	41	C1	Collecting exercise and health data	18
			C2	Assessing and diagnosing student data	15
			C3	Constructing student health profiles	8
D	Digital ethics and safety in Physical Education Teaching	34	D1	Ensuring educational equity in digital environments	12
			D2	Observing data ethics and protecting privacy	7
			D3	Observing digital copyright regulations	10
			D4	Warning of and monitoring students’ exercise-related risks	5
E	Professional activities	45	E1	Reflecting on digital teaching practices	20
			E2	Conducting cross-temporal and cross-spatial collaborative professional learning	8
			E3	Using digital technologies for learning and innovation	17
F	Cultivating students’ use of digital technologies to maintain health	22	F1	Evaluating digital resources	9
			F2	Using digital technologies to design health plans	7
			F3	Monitoring and regulating health behaviours	6
G	Personal characteristics for applying digital technologies	40	G1	Willingness to explore	18
			G2	Professional confidence	10
			G3	Enthusiasm for application	9
			G4	Digital resilience	3

Reference frequency refers to the number of independent quotations extracted from and coded in the original data for each category. It reflected how frequently a given dimension was mentioned in participants’ descriptions and served as an indicator of its relative prominence.

During selective coding, “digital competence among PSPETs” was established as the core category that integrated all other categories. Based on the original quotations and the exploratory findings, digital competence among PSPETs was defined as an integrated set of behaviours and attitudes developed during the stage of professional identity to prepare PSPETs for future digital physical education environments. It was manifested in the behavioural patterns and sustained willingness to use digital technologies throughout the processes of physical education planning, implementation, assessment, and professional development.

**Fig. 1:**
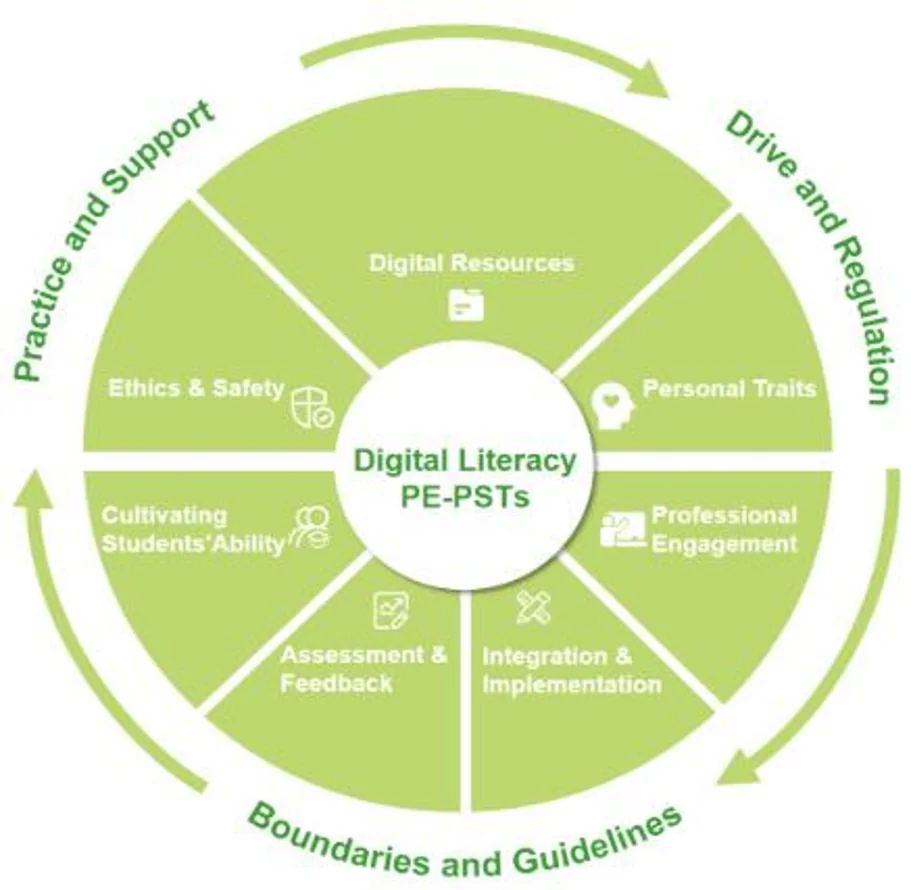
Storyline of digital competence among PSPETs.

A coherent storyline was constructed around this core category to clarify the theoretical relationships among the categories. Digital competence among PSPETs was conceptualized as a multilayered and dynamically interconnected structure (Fig. 1). Specifically, the core practice layer comprised digital resources for physical education teaching (A), teaching integration and implementation (B), and teaching assessment and feedback. These dimensions were the most prominent and concentrated topics in participants’ accounts and discussions. Professional activities (E) functioned as a key support process that drove the ongoing development and improvement of the core practices. Digital ethics and safety (D) emerged as an essential boundary and behavioural code for all digital practices, reflecting PSPETs’ initial but critical awareness of ethical issues. Cultivating students’ use of digital technologies to maintain health (F) represented the ultimate educational purpose of digital competence. Finally, the elements of personal characteristics for applying digital technologies (G), such as willingness to explore and digital resilience, played important moderating and motivating roles in responding to technological challenges and stimulating innovative applications.

### Validation of the digital competence framework for PSPETs

A cross-validation procedure was used. The 1,437 valid responses were randomly divided using SPSS 29.0 into an EFA subsample (*n* = 719) and a CFA subsample (*n* = 718). The two subsamples showed no apparent differences and were considered suitable for subsequent analyses. Item analysis was conducted using the total sample (*n* = 1,437). All 32 items demonstrated excellent discrimination. All independent-samples t tests were highly significant (*p* < .001), Cohen’s d values exceeded 1.40, and item-total correlations were greater than 0.48. Q16 was a reverse-scored item, whereas all other items showed positive discrimination. All items were therefore retained for subsequent analyses.

The total scale demonstrated high internal consistency, with Cronbach’s alpha of α = 0.939. The reliability coefficients for the seven dimensions ranged from 0.853 to 0.923. These results indicated reliable internal consistency.

#### Exploratory factor analysis

An EFA was first conducted using the EFA subsample (*n* = 719). The Kaiser–Meyer–Olkin measure was 0.950, and Bartlett’s test of sphericity was significant (χ² = 17,461.12, *p* < .001), indicating that the data were suitable for factor analysis. PCA with Varimax rotation was conducted. Based on Parallel Analysis and inspection of the scree plot, seven factors with eigenvalues greater than 1 were extracted. These factors explained 77.1% of the total variance (see Supplementary Material 1).

The rotated factor-loading matrix showed that all items loaded above 0.70 on their anticipated dimensions and displayed no substantial cross-loadings. These results provided preliminary support for the seven-factor structure (see Supplementary Material 1).

#### Confirmatory factor analysis

A CFA was conducted using the CFA subsample (*n* = 718) to test the theoretically specified seven-factor model. The fit indices indicated a good fit between the data and the model: χ²/df = 2.080, CFI = 0.973, TLI = 0.969, RMSEA = 0.039 (90% CI [0.035, 0.042]), and SRMR = 0.040. All standardized factor loadings (λ) were significant at *p* < .001 and ranged from 0.77 to 0.88 (Table 3). The path diagram for the CFA model is provided in Supplementary Material 1.

**Table 3:** Standardized factor loadings from the CFA of the Questionnaire on Digital Competence among PSPETs (*n* = 718).

Factor	Measurement item	Unstandardized loading	Standard error	z	*p*	Standardized loading	SMC
F1	Q1	1.000	–	–	–	0.849	0.720
F1	Q2	1.087	0.039	28.108	0.000	0.839	0.705
F1	Q3	1.047	0.036	29.201	0.000	0.859	0.738
F1	Q4	1.093	0.040	27.556	0.000	0.829	0.688
F1	Q5	1.042	0.038	27.764	0.000	0.833	0.694
F2	Q6	1.000	–	–	–	0.809	0.654
F2	Q7	1.092	0.043	25.436	0.000	0.828	0.686
F2	Q8	1.106	0.045	24.857	0.000	0.814	0.663
F2	Q9	1.200	0.046	26.194	0.000	0.846	0.715
F2	Q10	1.254	0.046	27.030	0.000	0.865	0.748
F3	Q11	1.000	–	–	–	0.864	0.747
F3	Q12	1.060	0.036	29.333	0.000	0.859	0.739
F3	Q13	0.892	0.034	26.525	0.000	0.807	0.652
F3	Q14	0.941	0.034	27.632	0.000	0.828	0.686
F4	Q15	1.000	–	–	–	0.878	0.771
F4	Q16	− 1.029	0.033	31.609	0.000	− 0.860	0.739
F4	Q17	0.977	0.034	28.782	0.000	0.818	0.669
F4	Q18	0.954	0.032	29.894	0.000	0.835	0.697
F4	Q19	1.076	0.034	31.266	0.000	0.855	0.731
F4	Q20	0.959	0.032	29.645	0.000	0.831	0.691
F5	Q21	1.000	–	–	–	0.857	0.734
F5	Q22	1.122	0.040	27.848	0.000	0.846	0.715
F5	Q23	0.932	0.038	24.300	0.000	0.773	0.597
F5	Q24	1.029	0.038	27.376	0.000	0.836	0.699
F6	Q25	1.000	–	–	–	0.844	0.713
F6	Q26	0.971	0.042	23.037	0.000	0.794	0.630
F6	Q27	0.956	0.041	23.476	0.000	0.808	0.653
F7	Q28	1.000	–	–	–	0.849	0.721
F7	Q29	1.041	0.038	27.476	0.000	0.830	0.689
F7	Q30	1.041	0.037	28.051	0.000	0.840	0.706
F7	Q31	1.000	0.038	26.407	0.000	0.809	0.655
F7	Q32	1.106	0.040	27.971	0.000	0.839	0.704

F1 = digital resources for physical education teaching; F2 = integration and implementation of physical education teaching; F3 = assessment and feedback in physical education teaching; F4 = digital ethics and safety in Physical Education Teaching; F5 = professional activities; F6 = cultivating students’ use of digital technologies to maintain health; F7 = personal characteristics for applying digital technologies. A dash (–) indicates a reference item. Q16 was reverse-scored; All factor loadings were significant at *p* < .001.All factor loadings were significant at *p* < .001. SMC = Squared Multiple Correlation.

#### Convergent and discriminant validity

The AVE and CR values for each dimension were calculated to assess construct validity (Table 4). The AVE values ranged from 0.67 to 0.72 and exceeded the threshold of 0.50 for all dimensions. The CR values ranged from 0.86 to 0.92 and exceeded the recommended criterion of 0.70. These findings indicated good convergent validity.

The square root of the AVE for each dimension was greater than its correlations with the other dimensions, satisfying the Fornell–Larcker criterion and supporting the discriminant validity of the scale^59^.

**Table 4:** Validity results for the Questionnaire on Digital Competence among PSPETs (*n* = 718).

Dimension	CR	AVE	1	2	3	4	5	6	7
F1	0.92	0.71	**0.84**						
F2	0.92	0.69	0.48	**0.83**					
F3	0.91	0.71	0.40	0.57	**0.84**				
F4	0.87	0.72	0.40	0.42	0.34	**0.85**			
F5	0.90	0.69	0.53	0.51	0.44	0.37	**0.83**		
F6	0.86	0.67	0.46	0.41	0.60	0.40	0.53	**0.82**	
F7	0.92	0.70	0.61	0.45	0.51	0.34	0.56	0.50	**0.84**

Bold diagonal values represent the square roots of the AVE values. Off-diagonal values represent correlations between factors; all correlations were significant at *p* < .001.

### Multiple regression analysis of digital competence among PSPETs and background variables

#### Common method bias

Harman’s Single-Factor Test was conducted using the full Phase 3 sample (*N* = 3,128) to assess CMB. An unrotated PCA was performed on all 32 items. As shown in Table 5, seven factors with eigenvalues greater than 1 were extracted. The first factor explained 41.00% of the variance, which was below the 50% threshold. This result indicated that CMB did not pose a serious threat to the validity of the study.

**Table 5:** Results of Harman’s Single-Factor Test.

Factor	Eigenvalue	Variance explained (%)	Cumulative variance explained (%)
1	13.12	41.00	41.00
2	2.84	8.88	49.88
3	2.15	6.72	56.60
4	1.78	5.56	62.16
5	1.42	4.44	66.60
6	1.28	4.00	70.60
7	1.03	3.22	73.82
8–32	–	< 3.00	–

#### Measurement invariance

The results of the measurement-invariance tests across the seven demographic variables are presented in Supplementary Material 1. The Configural Invariance models showed good fit across all groups, with CFI values ranging from 0.983 to 0.990 and RMSEA values ranging from 0.018 to 0.022. After equality constraints were imposed on the factor loadings and intercepts, the absolute values of ΔCFI did not exceed 0.006 (M2 → M3), and the absolute values of ΔRMSEA did not exceed 0.004. All results met the recommended criteria of ΔCFI ≥ − 0.010 and ΔRMSEA ≤ 0.015, supporting Scalar Invariance.

#### Main and interaction effects

The total digital competence score was specified as the dependent variable, and 12 predictor variables were entered into the regression model. The overall model was significant, F(12, 3115) = 90.59, *p* < .001, R² = 0.259, adjusted R² = 0.256. The VIF values ranged from 1.002 to 1.815, indicating no serious multicollinearity. The Durbin–Watson Statistic was 2.031, suggesting no significant residual autocorrelation.

As shown in Table 6, institutional level, GPA ranking, and academic year were the strongest positive correlates of digital competence. Students enrolled at 985 universities (β = 0.272, *p* < .001, Cohen’s f² = 0.095) and 211 universities (β = 0.162, *p* < .001, Cohen’s f² = 0.034) had significantly higher digital competence scores than students enrolled at ordinary undergraduate institutions. Students ranked in the top one-third of their GPA distribution (β = 0.314, *p* < .001, Cohen’s f² = 0.073) and the middle one-third (β = 0.123, *p* < .001, Cohen’s f² = 0.011) scored higher than those in the bottom one-third. Master’s students in the early stage (β = 0.228, *p* < .001, Cohen’s f² = 0.056) and advanced stage (β = 0.198, *p* < .001, Cohen’s f² = 0.045) scored higher than undergraduate students in the early stage. In addition, attendance at a first-tier city institution (β = 0.176, *p* < .001, Cohen’s f² = 0.035) and male gender (β = 0.075, *p* < .001, Cohen’s f² = 0.008) were associated with higher digital competence scores.

By contrast, low-income family status (β = − 0.106, *p* < .001, Cohen’s f² = 0.015) and First-Generation College Students status (β = − 0.102, *p* < .001, Cohen’s f² = 0.014) were significant negative correlates.

In terms of effect size, attendance at a 985 university (f² = 0.095) and ranking in the top one-third of the GPA distribution (f² = 0.073) had the strongest substantive effects, approaching a medium effect. Being a master’s student in the early stage (f² = 0.056), being a master’s student in the advanced stage (f² = 0.045), attending an institution in a first-tier city (f² = 0.035), and attending a 211 university (f² = 0.034) showed small effects. The effects of the remaining variables were negligible (f² ≤ 0.015), with gender showing the smallest effect (f² = 0.008).

**Table 6:** Multiple linear regression analysis of digital competence among PSPETs (*N* = 3,128).

Predictor (reference group)	B	SE	β	t	*p*	VIF	f²
Intercept	97.30	0.99	–	98.21	< 0.001	—	–
Male gender *(female)*	2.88	0.60	0.075	4.84	< 0.001	1.002	0.008
First-Generation College Students *(no)*	− 3.91	0.59	− 0.102	− 6.59	< 0.001	1.006	0.014
Low-income family *(no)*	− 4.62	0.67	− 0.106	− 6.87	< 0.001	1.006	0.015
985 university *(ordinary undergraduate institution)*	16.17	0.94	0.272	17.20	< 0.001	1.050	0.095
211 university *(ordinary undergraduate institution)*	7.19	0.70	0.162	10.27	< 0.001	1.052	0.034
Advanced-stage undergraduate *(early-stage undergraduate)*	4.86	0.71	0.120	6.83	< 0.001	1.305	0.015
Early-stage master’s student *(early-stage undergraduate)*	10.88	0.83	0.228	13.15	< 0.001	1.262	0.056
Advanced-stage master’s student *(early-stage undergraduate)*	12.32	1.04	0.198	11.85	< 0.001	1.171	0.045
Top one-third GPA ranking *(bottom one-third)*	12.55	0.83	0.314	15.09	< 0.001	1.815	0.073
Middle one-third GPA ranking *(bottom one-third)*	4.76	0.80	0.123	5.94	< 0.001	1.814	0.011
First-tier city *(third-tier city or below)*	8.91	0.85	0.176	10.49	< 0.001	1.183	0.035
Second-tier city *(third-tier city or below)*	2.09	0.65	0.054	3.21	0.001	1.180	0.003
R²=0.259							
Adjusted R²=0.256							
F(12, 3115) = 90.59, *p* < .001							
Durbin-Watson = 2.031.							

B = unstandardized regression coefficient; SE = standard error; β = standardized regression coefficient; VIF = Variance Inflation Factor; f² = Cohen’s local effect size (f² ≥ 0.020, small; f² ≥ 0.150, medium; f² ≥ 0.350, large).

As shown in Table 7, the ΔR² values for the low-income family × institutional level and First-Generation College Students × institutional level interaction terms were both below 0.1%. Neither F test was significant (*p* = .363 and *p* = .159, respectively), indicating that the predictive effect of institutional level on digital competence did not vary according to family background.

**Table 7:** Tests of the interaction effects between institutional level and family capital.

Interaction term	ΔR²	F	*p*
Low-income family × institutional level	0.001	1.014	0.363
First-Generation College Students × institutional level	0.001	1.843	0.159

ΔR² represents the increase in R² from the interaction model relative to the main-effects model (R²=0.259, adjusted R²=0.256). Neither F test for the two interaction terms reached statistical significance (*p* > .05), and both ΔR² values were below 0.1%. The conclusions remained unchanged after Bonferroni correction (α′ = 0.025).

## Discussion

This study addressed the constituent dimensions of a digital competence framework for PSPETs, the development and validation of a corresponding measurement instrument, and group differences in digital competence. The three-stage empirical investigation showed that: (1) the digital competence framework for PSPETs comprised seven main dimensions—digital resources for physical education teaching, integration and implementation of physical education teaching, assessment and feedback in physical education teaching, digital ethics and safety in Physical Education Teaching, professional activities, cultivating students’ use of digital technologies to maintain health, and personal characteristics for applying digital technologies—which were further divided into 25 subdimensions; (2) the first-order CFA model demonstrated a good fit (χ²/df = 2.08, CFI = 0.973, TLI = 0.969, RMSEA = 0.039, AVE = 0.67–0.72, CR = 0.86–0.92), indicating good internal consistency and supporting the construct validity and reliability of both the proposed PSPET-DC framework and its measurement instrument; and (3) higher academic performance, more advanced stages of study, attendance at higher-level institutions or institutions located in more developed cities, and more advantaged family backgrounds were positively associated with higher digital competence scores, whereas First-Generation College Students status and low-income family background were negatively associated with digital competence.

To examine the similarities and differences between the proposed PSPET-DC framework and mainstream general frameworks, Supplementary Material 1 functionally aligned its first-level dimensions with the European Union’s DigCompEdu framework, the UNESCO ICT Competency Framework for Teachers Version 3 (UNESCO ICT-CFT V3), the International Society for Technology in Education (ISTE) Standards for Educators, and China’s Teachers’ Digital Literacy standard. Four dimensions—digital resources for physical education teaching (A), integration and implementation of physical education teaching (B), assessment and feedback in physical education teaching (C), and professional activities (E)—showed functional correspondences with the general frameworks. This finding confirms the cross-disciplinary universality of core teaching processes, including lesson preparation, instruction, assessment, and reflection. It suggests that subject-specific digital competence frameworks respond to particular practical needs while retaining a common foundation. Similar efforts in science and English language education support this view^11,12^.

Nevertheless, although the functional mappings between dimensions were similar, the grounded-theory analysis assigned these dimensions meanings that were specific to physical education. For example, “assessment and feedback” (C) in general frameworks generally refers to academic assessment and feedback on assignments^29,30^. By contrast, subdimensions such as “collecting exercise and health data” and “constructing student health profiles” in the present framework directly address practices specific to physical education, including monitoring exercise load and tracking students’ physical fitness and health. The meaning of digital ethics and safety (D) is also substantially expanded in the physical education context. When teachers collect students’ physiological data, including heart rate, body morphology, athletic ability, and health conditions, they must simultaneously address privacy protection and the monitoring of exercise-related risks. These concerns are not adequately covered by general frameworks or by frameworks developed for predominantly knowledge-based subjects^12^.

Although these concepts have rarely emerged in existing studies of physical education teachers’ digital competence^32–34^, they have been extensively addressed in empirical studies of digital technology use in physical education classes. Examples include wearable devices, exercise-load monitoring and real-time feedback, and video-based movement analysis^13,21^, as well as emerging technologies such as AI-based visual recognition, motion capture, and AI-assisted movement assessment^24^. Nearly all of these practices correspond to specific competence dimensions in the present framework. This suggests that subject-specific frameworks may provide a more precise conceptual foundation and measurement tool for developing teachers’ digital competence in particular disciplines. Their practical effectiveness, however, requires further empirical examination.

In addition, the dimensions of cultivating students’ use of digital technologies to maintain health (F) and personal characteristics for applying digital technologies (G) had no independent counterparts in the four general frameworks. Falloon (2020), however, argued when extending the TPACK framework that teachers’ digital competence includes attitudinal elements required for effective, safe, and productive technology use. Previous research has also incorporated personality-related dimensions into competence structures^32–34^, while frameworks developed for other subjects have included dimensions that intersect subject-specific goals with digital technology^11,12^.

Dimension F is related to the emphasis in DigCompEdu on fostering learners’ digital competence, but DigCompEdu focuses on general digital competence, whereas the present framework emphasizes guiding students in using digital tools for health self-management. The TPACK framework highlights the deep integration of technological, pedagogical, and content knowledge^60^. Physical education, meanwhile, has the central mission of promoting physical activity and lifelong autonomous health management^61^. Therefore, helping students use digital technologies for health self-monitoring and planning represents a natural extension of physical education content knowledge in the digital age. Yang and Shi (2026) similarly noted that physical education teachers have a distinctive responsibility to connect students’ physical activity with complex digital health data^62^.

With the support of wearable devices and mobile applications, students can transform abstract health concepts into trackable personal data and continuously attend to and regulate their health in authentic contexts^63^. In the long term, this may support autonomous development and health management in a digital society. However, this outcome depends particularly on teachers’ ability to guide students in critically interpreting health data^64^.

Dimension G resembles the attitudinal aspect of digital awareness in China’s standard. However, the Chinese standard does not incorporate these elements as an independent competence structure, whereas the present framework does so through dimension G. The Technology Acceptance Model and the UTAUT framework have long identified perceived usefulness and perceived ease of use as key antecedents of technology adoption^65–67^. In physical education environments, which are often outdoors, highly dynamic, and noisy, technological confidence and willingness to explore may be particularly important^68^. As one participant stated:“The digital tools used in physical education classes are generally quite complex … Without interest, teachers are unlikely to use them proactively because the process is too cumbersome” (P7—11, PST).

Overall, the proposed PSPET-DC framework follows fundamental teaching principles and retains the core structure shared across disciplines. At the same time, it operationalizes a subject-specific perspective by incorporating the emergent dimensions F and G and contextualizing existing dimensions within physical education. In this respect, it provides a potential bridge between general digital competence frameworks and the practical demands of physical education teaching.

After controlling for the other variables, students at Project 985 universities scored, on average, 16.17 points higher in overall DC than students at ordinary universities (f² = 0.095). This difference is equivalent to approximately one-tenth of the scale range, confirming that institutional level is similarly associated with students’ DC in the Chinese educational context^69,70^. In fact, China’s Project 985 and Project 211 initiatives represent not only several-fold differences in per-student government funding^71^, but also the concentration of high-quality faculty, academic resources, and hardware facilities^72^. Previous research has indicated that access to equipment and digital role modelling by university lecturers significantly influence preservice teachers’ technology integration competence^9^. In addition, China’s National College Entrance Examination system conducts large-scale stratified selection based on academic ability^73^. Students admitted to key universities are therefore generally more academically capable, which helps explain why enrollment at Project 985 and Project 211 institutions emerged as a positive predictor of DC.

The association between academic performance and digital competence was also notable. The effect size for GPA ranking was f²=0.073, slightly exceeding that for academic stage (f² =0.045). Students in the top third of the GPA distribution scored, on average, 12.55 points higher than those in the bottom third. Existing evidence suggests that strong academic performance may reflect greater learning engagement and self-regulation^74^, both of which may be associated with the active exploration and effective use of digital technologies. Similarly, students with stronger self-regulated learning skills are more capable of using digital tools to plan their learning, search for resources, and monitor their progress^75^.PSPETs at the master’s level may have more opportunities than undergraduate students to encounter digital technologies through coursework, microteaching, and practicum experiences. In the present study, both early-stage and advanced-stage master’s students reported significantly higher digital competence than early-stage undergraduate students. This pattern may reflect the accumulation of experience associated with educational stage. However, because the data were cross-sectional, the causal direction of this association requires verification through longitudinal research.

The association between gender and digital competence was statistically significant (β = 0.075, *p* < .001), but the effect size was small (f² =0.008). This result differs from previous findings that PSPETs’ digital competence varies substantially by gender^70^. Women have often been considered less likely to accept and use technology because of factors such as technological self-efficacy and gender stereotypes at the sociocultural level^76^. Nevertheless, recent research and practice suggest that, with the increasing availability of teacher digital competence development and the implementation of more inclusive and targeted training programmes, access to digital technology training and development opportunities is becoming more equitable across genders^77,78^. The traditional gender digital divide may therefore gradually narrow as a result of institutionalized training coverage and more targeted interventions. This interpretation nevertheless requires verification using longitudinal data collected across different periods.

First-Generation College Students status (β= − 0.102, f²=0.014) and low-income family status (β = − 0.106, f²= 0.015) were negatively associated with digital competence. This finding further indicates that disparities in family capital and digital competence remain evident among PSPETs^79^. International research has shown that, in many countries, First-Generation College Students face greater limitations in digital access and are more likely to hold negative perceptions of the usefulness of digital technologies^80,81^.

Compared with many other countries, family ties in China are relatively close, and children’s educational development is strongly shaped by the family environment^82^. In this context, family-related barriers faced by First-Generation College Students—including limited access to basic tools and resources and disruptions in the learning environment—may affect their digital learning experiences and may even be more consequential than individual-level and university-related barriers^80,82^. These barriers may constrain the development of digital competence among First-Generation College Students.

By contrast, the negative association between low-income family status and digital competence is more directly related to limitations in economic capital. Such limitations may manifest as inadequate or absent hardware resources, including digital devices, internet access, and learning tools. Existing research suggests that these limitations cannot readily be addressed solely by providing access during the university period^83^.

Notably, the interaction between institutional level and family capital was not statistically significant. From an effect-size perspective, this finding indicates that the digital competence advantage associated with institutional level and the constraints associated with family background operate independently and do not moderate one another. Institutional level therefore neither strengthened nor weakened the association between family background and digital competence. Although higher-level universities may provide better access to equipment and teaching expertise, they may not immediately alter individuals’ early processes of technological socialization^84,85^. Students from more advantaged backgrounds may be more likely to view technology as a tool for exploration and innovation. In contrast, students with fewer family resources may have had fewer opportunities for exploratory digital practices in early life and may consequently experience greater cognitive load and technology anxiety^79^, which may be associated with lower digital competence scores.

## Conclusion

This study used an exploratory sequential mixed-methods design to construct and validate a theoretical framework and assessment scale for PSPETs’ digital competence. It then used a large-scale survey to examine the associations between PSPETs’ digital competence and several background factors. First, the digital competence framework broadly covered the structures and elements of physical education teaching and reflected the movement-based and contextually open nature of the subject. Second, the Questionnaire on Digital Competence among PSPETs developed from this framework demonstrated good psychometric properties and showed practical value for routine administration in physical education teacher education programmes in China. Finally, the large-scale survey indicated that institutional level, academic performance, academic stage, and institutional location were positively associated with PSPETs’ digital competence, whereas First-Generation College Students status and low-income family background were negatively associated with digital competence. The interaction between institutional level and family capital was not significant, suggesting that the effects of early technological socialization may operate relatively independently.

At the theoretical level, this study demonstrates the feasibility of developing a subject-specific digital competence framework for pre-service teachers and provides a potentially useful theoretical perspective for understanding the meaning and components of PSPETs’ professional competence in the digital age. Although the sample and data were drawn from the Chinese teacher education system, the competence dimensions identified in this study do not depend on the value orientations or institutional characteristics of a particular cultural group. Rather, they are grounded in the practical logic of physical education teaching.

Regardless of the educational system, physical education uses bodily practice as its primary medium, movement-skill acquisition as a central process, and health promotion as a major goal. These subject characteristics mean that physical education teaching shares broadly similar structures, processes, and purposes across cultural contexts. Accordingly, after appropriate localization, the present framework may be transferable to physical education teacher education contexts in other countries and regions. It may also provide a workable theoretical starting point for examining the framework’s cross-cultural robustness. In addition, the PSPET-DC framework developed through an exploratory sequential mixed-methods design offers a replicable methodological approach for developing subject-specific digital competence frameworks for pre-service teachers in other disciplines.

At the practical level, the dimensions identified in the framework can serve as a reference for curriculum developers when setting learning objectives and selecting course content. Curriculum developers can review existing curricula, identify weak or missing content areas, and add targeted modules. The Questionnaire on Digital Competence among PSPETs can also function as a convenient tool for assessing curriculum needs and evaluating curriculum outcomes, thereby providing empirical evidence for curriculum-related decision-making.

For teacher educators, the emergence of the personal characteristics dimension indicates that the development of PSPETs’ digital competence should not be reduced to technical skills training. Emotional and motivational factors should also be addressed. Low-risk experiential activities, peer modelling, and reflective practice may gradually help PSPETs develop positive orientations toward the use of digital technologies.

Higher education institutions could reduce disparities in hardware provision and modelling expertise across institutions by sharing digital teaching case repositories and establishing cross-institutional virtual professional learning communities. They could also establish peer-mentoring systems in which preservice teachers with stronger digital competence support students in earlier stages of training, as well as equipment-lending libraries to lower material access barriers. Moving the development of PSPETs’ digital competence from fragmented technical training toward subject-integrated preparation may help reduce digital competence disparities associated with resource allocation and family background during the pre-service stage.

## Limitations and future directions

First, this study used cross-sectional data. The findings therefore reflected statistical associations between variables rather than causal relationships. Future longitudinal research could further clarify the developmental trajectory of digital competence and examine the direction of the observed associations.

Second, the data were collected using a self-report scale and may have been affected by social desirability. Although Harman’s Single-Factor Test indicated that common method bias was not serious, self-report measures have inherent limitations. Future research should incorporate performance-based tasks situated in authentic teaching contexts to cross-validate self-reported competence.

Third, all participants were drawn from the Chinese higher education system. The applicability of the findings to other educational systems and cultural contexts therefore remains to be established. Cross-cultural comparative studies could assess the framework’s robustness across contexts.

Fourth, AI, virtual reality (VR), and multimodal wearable devices are rapidly entering physical education. The current operational items do not yet fully reflect these emerging technologies. Although such technologies remain relatively uncommon in routine teaching practice, future research could build on the existing seven-factor structure by developing dedicated subdimensions for AI-assisted movement assessment and VR-based contextual simulation.

Fifth, digital technologies evolve rapidly, and new teaching tools continue to emerge. Any framework constructed on the basis of the current technological ecosystem will have difficulty fully anticipating changes in future teaching contexts. Future studies should therefore update and refine the framework on a timely basis to preserve its ecological validity and instructional value.

## Supplementary Information

Below is the link to the electronic supplementary material.

Supplementary Material 1

## Data Availability

The data that support the findings of this study are available from the corresponding author upon reasonable request.
