# Supplementary material for: Development and validation of the digital competence framework and scale for pre-service physical education teachers
Source: Sci Rep. 2026 Jul 23;16:26807. doi: 10.1038/s41598-026-63030-z (PMC13518990; doi:10.1038/s41598-026-63030-z)
Supplement: Supplementary file 1 [file 41598_2026_63030_MOESM1_ESM.docx]

**Supplementary Material**

*Supplementary Material 1. Digital Competence Survey Scale for Pre-service Physical Education Teachers (PSPETs)*

| Item | Strongly Agree | Agree | Neutral | Disagree | Strongly Disagree |
| --- | --- | --- | --- | --- | --- |
| I can skillfully use online platforms to find the teaching resources I need. |  |  |  |  |  |
| I can evaluate the scientific validity and safety of online teaching resources in terms of movement demonstrations and instructional arrangements. |  |  |  |  |  |
| I have developed my own systematic methods for organizing and preserving the digital teaching resources I have accumulated. |  |  |  |  |  |
| I can use video-editing software to produce short videos demonstrating physical movements that include subtitles and narration. |  |  |  |  |  |
| I can use text and image editing tools to create engaging diagrams for explaining sports principles or tactics. |  |  |  |  |  |
| Before designing a lesson plan, I use digital tools to understand students’ interests in physical activity and their existing skill levels. |  |  |  |  |  |
| When designing a physical education lesson, I actively consider the teaching stages in which digital technologies can be integrated most effectively to achieve the instructional objectives. |  |  |  |  |  |
| I can use digital technologies to design a high-quality lesson plan. |  |  |  |  |  |
| During simulated teaching or teaching practicums, I can skillfully operate multimedia equipment and teaching software to complete teaching demonstrations. |  |  |  |  |  |
| I can use classroom interaction software or miniature wearable devices to conduct classroom management activities, such as assigning group tasks, timing activities, and randomly calling on students. |  |  |  |  |  |
| I can use a variety of digital tools, such as fitness watches and heart-rate monitors, to collect students’ exercise and health data. |  |  |  |  |  |
| I can interpret students’ post-exercise data reports and determine whether their exercise load is appropriate. |  |  |  |  |  |
| Based on students’ exercise and health data, I can provide specific and actionable recommendations or adjustment plans for improvement. |  |  |  |  |  |
| I know how to integrate information such as students’ physical fitness data and exercise habits to create an initial visual health profile for each student. |  |  |  |  |  |
| When arranging the use of digital devices such as tablets and sensors, I ensure that all students have equal opportunities to operate the devices and participate in the experience. |  |  |  |  |  |
| I believe that using students’ exercise data for instructional analysis without their explicit consent is acceptable if it improves classroom efficiency. |  |  |  |  |  |
| If I need to present a student’s exercise data in a public setting, I will anonymize the data beforehand. |  |  |  |  |  |
| When creating teaching materials or videos, I consciously use authorized materials or materials identified as available for public use, and I properly acknowledge the sources. |  |  |  |  |  |
| When organizing high-intensity exercise, I recognize the importance of using real-time monitoring of heart rate and other data to provide early warnings of exercise-related risks. |  |  |  |  |  |
| I can give examples of how digital technologies can be used to prevent students from experiencing accidents during exercise. |  |  |  |  |  |
| After class, I review recordings of my teaching to reflect on the appropriateness and actual effectiveness of my use of digital technologies. |  |  |  |  |  |
| I am willing and able to exchange experiences and insights regarding digital teaching with other physical education teachers through online meetings, professional communities, and similar channels. |  |  |  |  |  |
| I frequently use online platforms to learn about cutting-edge physical education teaching concepts and examples of digital applications from China and abroad. |  |  |  |  |  |
| When I learn about a new digital technology, I actively consider its potential applications and possible uses in physical education teaching contexts. |  |  |  |  |  |
| I can teach students how to judge the scientific validity and reliability of the content provided by an exercise- or fitness-related application or website. |  |  |  |  |  |
| I can guide students in using online technologies to develop personalized exercise or dietary plans for themselves. |  |  |  |  |  |
| I can teach students how to reasonably adjust their lifestyle habits based on activity levels and sleep data provided by fitness bands. |  |  |  |  |  |
| I believe that the appropriate use of digital technologies can effectively address some problems in traditional physical education teaching. |  |  |  |  |  |
| I actively follow the latest trends in the application of digital technologies in physical education. |  |  |  |  |  |
| I believe that I am capable of learning and mastering the various digital tools that may be required for physical education teaching in the future. |  |  |  |  |  |
| I am highly enthusiastic and curious about trying to introduce new digital products into physical education classes. |  |  |  |  |  |
| When solving technical problems involving digital teaching tools, I demonstrate considerable patience and persistence and do not give up easily. |  |  |  |  |  |

*Supplementary Material 2. Distribution of Demographic Characteristics of the Samples in Stages 2 and 3*

| Variable | Category | Stage2 (N=1437) | | Stage3 (N=3128) | |
| --- | --- | --- | --- | --- | --- |
|  |  | n | % | n | % |
| Gender | Male | 725 | 50.45 | 1723 | 55.09 |
|  | Female | 712 | 49.55 | 1405 | 44.91 |
| First-Generation College Students | No | 848 | 59.01 | 1573 | 50.29 |
|  | Yes | 589 | 40.99 | 1555 | 49.71 |
| Low-Income Family | No | 1072 | 74.60 | 2301 | 73.56 |
|  | Yes | 365 | 25.40 | 827 | 26.44 |
| Institutional Level | Ordinary undergraduate institution | 867 | 60.33 | 1978 | 63.23 |
|  | 211 university (non-985) | 366 | 25.47 | 782 | 25.00 |
|  | 985 university | 204 | 14.20 | 368 | 11.76 |
| Academic Year | Early-stage undergraduate | 522 | 36.33 | 1095 | 35.01 |
|  | Advanced-stage undergraduate | 552 | 38.41 | 1071 | 34.24 |
|  | Early-stage master’s student | 235 | 16.35 | 631 | 20.17 |
|  | Advanced-stage master’s student | 128 | 8.91 | 331 | 10.58 |
| GPA Ranking  (Grade Point Average Ranking) | Top third | 583 | 40.57 | 1114 | 35.61 |
|  | Middle third | 501 | 34.86 | 1397 | 44.66 |
|  | Bottom third | 353 | 24.57 | 617 | 19.73 |
| City Tier | First-tier city | 289 | 20.11 | 541 | 17.30 |
|  | Second-tier city | 529 | 36.81 | 1306 | 41.75 |
|  | Third-tier city or below | 619 | 43.08 | 1281 | 40.95 |

*Note: The operational definitions of the variables in the table are as follows. Gender was coded with “female” as the reference category. First-Generation College Students refers to students whose parents' highest educational attainment is high school or below. Low-Income Family refers to students receiving national financial aid for students from impoverished families. This policy provides an annual subsidy of RMB 2,000–4,000 to financially disadvantaged students enrolled in higher education institutions in China. Institutional level was classified according to the official classification of the Ministry of Education of China, with 985 universities representing the highest level, followed by 211 universities and then ordinary undergraduate institutions. For academic year, “early-stage undergraduate” includes first- and second-year undergraduate students, “advanced-stage undergraduate” includes third- and fourth-year undergraduate students, “early-stage master’s student” refers to first-year master’s students, and “advanced-stage master’s student” refers to second-year master’s students or above. The city tiers of the institutions’ locations were classified according to the criteria proposed by the China Institute of City Research, based on commercial attractiveness and the level of economic development. GPA Ranking refers to students’ relative academic standing within their class or major. These data were uniformly extracted from the academic administration system by class counselors.*

*Supplementary Material 3. Dummy-Variable Coding Scheme for Categorical Variables*

| Variable | Original Categories | Reference Category | Dummy Variable | Coding  (1 = Yes, 0 = No) |
| --- | --- | --- | --- | --- |
| Gender | Male, Female | Female | Male | Male=1, Female = 0 |
| First-Generation College Students | Yes, No | No | First-Generation College Students | Yes = 1, No = 0 |
| Low-Income Family | Yes, No | No | Low-Income Family | Yes = 1, No = 0 |
| Institutional Level | 985 university /  211 university /  Ordinary undergraduate institution | Ordinary undergraduate institution | 985 university | 985 university=1,  other = 0 |
|  |  |  | 211 university | 211 university=1,  other = 0 |
| Academic Year | Early-stage undergraduate /advanced-stage undergraduate  / early-stage master’s student  / advanced-stage master’s student | Early-stage undergraduate | Advanced-stage undergraduate | Advanced-stage undergraduate= 1,  other = 0 |
|  |  |  | Early-stage master’s student | Early-stage master’s student = 1,  other = 0 |
|  |  |  | Advanced-stage master’s student | Advanced-stage master’s student=1,  other = 0 |
| GPA Ranking | Top third / middle third / bottom third | Bottom third | Top third | Top third = 1,  other = 0 |
|  |  |  | Middle third | Middle third = 1,  other = 0 |
| City Tier of Institution | First-tier city / second-tier city / third-tier city or below | Third-tier city or below | First-tier city | First-tier city = 1,  other = 0 |
|  |  |  | Second-tier city | Second-tier city=1,  other = 0 |

*Supplementary Material 4. Overall Table of the Open Coding and Categorization Process*

| No. | Initial Concept |
| --- | --- |
| OC-001 | Searching for physical education teaching demonstration videos on video platforms |
| OC-002 | Browsing physical education teaching cases recommended by social media |
| OC-003 | Obtaining courseware and lesson-plan templates from academic resource platforms |
| OC-004 | Reviewing demonstration course content on online course platforms |
| OC-005 | Browsing feature articles on physical education promoted by industry media |
| OC-006 | Searching for illustrated materials explaining key points of sports techniques |
| OC-007 | Searching for digital materials on the rules and officiating methods of sports |
| OC-008 | Judging the technical accuracy of movements shown in videos |
| OC-009 | Distinguishing the scientific validity and authority of online teaching resources |
| OC-010 | Selecting digital content suitable for different educational stages |
| OC-011 | Assessing the operability and applicability of digital resources |
| OC-012 | Classifying and storing digital files by teaching unit |
| OC-013 | Establishing a personal physical education teaching resource repository |
| OC-014 | Tagging and categorizing digital materials |
| OC-015 | Backing up and synchronizing teaching resources using cloud storage |
| OC-016 | Editing and producing short videos demonstrating sports movements |
| OC-017 | Designing digital lesson plans that include demonstrations breaking movements down into steps |
| OC-018 | Recording students’ exercise performance for instructional analysis |
| OC-019 | Producing courseware integrating text, images, and videos |
| OC-020 | Designing interactive digital assessment tools |
| OC-021 | Using questionnaire tools to investigate students’ interests in physical activity and their foundations |
| OC-022 | Analyzing historical physical fitness test data to track trends in students’ physical development |
| OC-023 | Using pre-class diagnostic tests to determine students’ level of skill mastery |
| OC-024 | Designing technology-integrated classroom training plans |
| OC-025 | Designing digital tool-supported independent exercise tasks after class |
| OC-026 | Designing instructional activities involving technical comparisons using video analysis |
| OC-027 | Designing activities to regulate exercise load in conjunction with wearable devices |
| OC-028 | Designing physical education knowledge quizzes using interactive feedback platforms |
| OC-029 | Designing differentiated teaching tasks based on differences in student data |
| OC-030 | Dynamically adjusting exercise intensity during class based on real-time data |
| OC-031 | Using digital display devices to explain movements step by step |
| OC-032 | Organizing students to engage in independent practice using digital tools |
| OC-033 | Presenting the results of technical comparison and analysis using digital display devices |
| OC-034 | Using multimedia signals to organize the pace of classroom activities |
| OC-035 | Completing attendance records using an electronic system |
| OC-036 | Using interactive tools to obtain students’ real-time feedback |
| OC-037 | Using digital tools to track students’ participation in classroom physical activities |
| OC-038 | Using management tools for grouping and task assignment |
| OC-039 | Collecting heart-rate and exercise-load data |
| OC-040 | Quantifying technical movement data through video playback |
| OC-041 | Recording physical fitness test results using electronic devices |
| OC-042 | Collecting digital records of students’ exercise check-ins |
| OC-043 | Analyzing data to identify deviations in students’ technical movements |
| OC-044 | Tracking individual student progress using data comparisons |
| OC-045 | Assessing whether heart-rate data have reached the target range |
| OC-046 | Analyzing data to determine the extent to which the class has achieved its overall objectives |
| OC-047 | Integrating multidimensional data to generate visual health reports for students |
| OC-048 | Incorporating formative assessment data into students’ health records |
| OC-049 | Paying attention to inequalities in participation caused by differences in technological conditions |
| OC-050 | Designing alternative arrangements to ensure the participation of students without access to devices |
| OC-051 | Avoiding the exacerbation of differences in students’ exercise performance through the use of technology |
| OC-052 | Preventing the risk of students’ health data being leaked |
| OC-053 | Obtaining informed consent from parents for the collection of student data |
| OC-054 | Not uploading student videos or images to unofficial platforms |
| OC-055 | Acknowledging sources when using online materials |
| OC-056 | Avoiding the unauthorized reproduction of others’ works |
| OC-057 | Understanding and complying with Creative Commons licenses |
| OC-058 | Using wearable devices to identify early warning signals of exercise-related risks |
| OC-059 | Detecting abnormal exercise loads through real-time monitoring |
| OC-060 | Reflecting on teaching through classroom video playback |
| OC-061 | Evaluating the effectiveness of technology integration using feedback from student data |
| OC-062 | Writing digital teaching journals to record experiences and shortcomings |
| OC-063 | Adjusting subsequent teaching strategies based on the results of reflection |
| OC-064 | Participating in collaborative lesson preparation through online teacher communities |
| OC-065 | Participating in interinstitutional teaching seminars through online platforms |
| OC-066 | Collaboratively developing teaching resources using online documents |
| OC-067 | Learning about emerging sports technology through online courses |
| OC-068 | Subscribing to academic resources to follow cutting-edge developments in the field |
| OC-069 | Participating in online professional development to improve digital teaching skills |
| OC-070 | Proactively applying emerging tools to teaching innovation |
| OC-071 | Guiding students in distinguishing the scientific validity of digital health tools |
| OC-072 | Guiding students in critically evaluating the safety of online fitness guidance |
| OC-073 | Developing students’ ability to judge health-related marketing information |
| OC-074 | Guiding students in using wearable devices to set exercise goals |
| OC-075 | Guiding students in developing personalized digital exercise plans |
| OC-076 | Guiding students to develop healthy habits through data tracking |
| OC-077 | Teaching students to interpret data and engage in self-regulation |
| OC-078 | Maintaining an open attitude toward unfamiliar digital tools |
| OC-079 | Being willing to invest time in learning new technologies |
| OC-080 | Being confident in solving common problems in technology integration |
| OC-081 | Having confidence in using technology to improve teaching effectiveness |
| OC-082 | Being enthusiastic about integrating technology into physical education teaching |
| OC-083 | Rapidly adjusting teaching strategies when equipment malfunctions |
| OC-084 | Proactively seeking assistance from peers when encountering technological difficulties |

*Supplementary Material 5. Correspondence Between Representative Quotations and Initial Concepts for Each Main Category*

| Main Category | Representative Quotation | Corresponding Initial Concept |
| --- | --- | --- |
| A.Digital Resources for Physical Education Teaching | “When preparing lessons during my practicum, I searched Douyin for demonstration videos of inside-foot passing in soccer. I looked through several videos. Some of them used slow-motion replays, which made the movements quite clear, but the movements themselves were actually not very accurate.” (P03—08, PST) | OC-001 Searching for physical education teaching demonstration videos on video platforms  OC-008 Judging the technical accuracy of movements shown in videos |
| B. Integration and Implementation in Physical Education Teaching | “During my practicum, I put a heart-rate monitor on each student in the endurance-running class. I remember it very clearly: it was a 12-minute timed run. I stood beside the track and watched the real-time data on a tablet. Some students’ heart rates did not rise at all, which clearly indicated that they were slacking off, while other students’ heart rates rose above 180. I immediately called out to one of them to slow down.” (P07—04, PST) | OC-027 Designing activities to regulate exercise load in conjunction with wearable devices  OC-030 Dynamically adjusting exercise intensity during class based on real-time data |
| C. Assessment and Feedback in Physical Education Teaching | “When I taught a throwing lesson, I recorded each student’s movements. After recording them, I asked the students to watch the replays themselves and then provided feedback based on the replays. Later, I put the recordings from the first lesson together with those from several subsequent lessons for comparison, so each student could see how much they had improved.” (P18—03/04, IST) | OC-040 Quantifying technical movement data through video playback  OC-044 Tracking individual student progress using data comparisons |
| D.Digital Ethics and Safety in Physical Education Teaching | “We collect students’ physical fitness test data, heart-rate data, and some videos from the exercise process. If these materials were obtained by a third-party platform, parents would certainly have concerns.” (P18—07, PST) | OC-052 Preventing the risk of students’ health data being leaked |
| E.Professional Activities | “During my practicum, every time I finished teaching a class that used digital tools, I recorded the lesson and watched it again afterward. The most painful part was watching my first lesson and realizing that I had said a lot of unnecessary things.” (P05—11, PST) | OC-060 Reflecting on teaching through classroom video playback |
| F.Fostering Students’ Use of Digital Technology for Health Maintenance | “With digital technology now so widespread, we need to help students genuinely learn to manage their health through digital means. For example, my off-campus mentor has students record their activity using fitness bands, with the aim of enabling them to continue conveniently monitoring their bodies in the same way after they leave physical education classes.” (P14—04, PST) | OC-076 Guiding students to develop healthy habits through data tracking  OC-077 Teaching students to interpret data and engage in self-regulation |
| G.Personality Traits in Digital Technology Application | “Recently, I tried an artificial intelligence (AI) movement-analysis application. You perform a movement in front of the camera, and it immediately tells you what is wrong. I plan to assign homework next lesson so that students can use this application at home to do strength exercises.” (P20—01/P20—06, IST) | OC-079Being willing to invest time in learning new technologies |

*Note: The identifiers in the representative quotation table include the participant number (e.g., P06) and the sequence number of the original statement (e.g., 03), enabling the source of each quotation to be traced. PST = Pre-service Teacher; PETE = Physical Education Teacher Educator; IST = In-service Teacher. OC-XXX denotes an Initial Concept.*

*Supplementary Material 6. Variance Explained by the Digital Competence Framework for Pre-service Physical Education Teachers (PSPETs) (n=719)*

| Factor | Initial Eigenvalue | | | Extraction Sum of Squared Loadings | | | Rotation Sum of Squared Loadings | | |
| --- | --- | --- | --- | --- | --- | --- | --- | --- | --- |
|  | Eigenvalue | Variance Explained (%) | Cumulative Variance Explained (%) | Eigenvalue | Variance Explained (%) | Cumulative Variance Explained (%) | Eigenvalue | Variance Explained (%) | Cumulative Variance Explained (%) |
| 1 | 12.280 | 38.376 | 38.376 | 12.280 | 38.376 | 38.376 | 4.689 | 14.652 | 14.652 |
| 2 | 3.124 | 9.763 | 48.139 | 3.124 | 9.763 | 48.139 | 3.928 | 12.274 | 26.927 |
| 3 | 2.408 | 7.526 | 55.665 | 2.408 | 7.526 | 55.665 | 3.854 | 12.044 | 38.970 |
| 4 | 2.287 | 7.147 | 62.812 | 2.287 | 7.147 | 62.812 | 3.753 | 11.727 | 50.698 |
| 5 | 1.805 | 5.642 | 68.454 | 1.805 | 5.642 | 68.454 | 3.181 | 9.941 | 60.638 |
| 6 | 1.640 | 5.126 | 73.580 | 1.640 | 5.126 | 73.580 | 3.023 | 9.447 | 70.086 |
| 7 | 1.111 | 3.472 | 77.052 | 1.111 | 3.472 | 77.052 | 2.229 | 6.967 | 77.052 |
| 8 | 0.418 | 1.305 | 78.357 | - | - | - | - | - | - |
| 9 | 0.404 | 1.263 | 79.621 | - | - | - | - | - | - |
| 10 | 0.379 | 1.183 | 80.804 | - | - | - | - | - | - |
| 11 | 0.369 | 1.154 | 81.958 | - | - | - | - | - | - |
| 12 | 0.365 | 1.140 | 83.098 | - | - | - | - | - | - |
| 13 | 0.350 | 1.095 | 84.193 | - | - | - | - | - | - |
| 14 | 0.340 | 1.063 | 85.256 | - | - | - | - | - | - |
| 15 | 0.333 | 1.042 | 86.298 | - | - | - | - | - | - |
| 16 | 0.323 | 1.011 | 87.308 | - | - | - | - | - | - |
| 17 | 0.317 | 0.991 | 88.300 | - | - | - | - | - | - |
| 18 | 0.312 | 0.975 | 89.275 | - | - | - | - | - | - |
| 19 | 0.293 | 0.917 | 90.192 | - | - | - | - | - | - |
| 20 | 0.287 | 0.898 | 91.090 | - | - | - | - | - | - |
| 21 | 0.281 | 0.878 | 91.968 | - | - | - | - | - | - |
| 22 | 0.268 | 0.837 | 92.805 | - | - | - | - | - | - |
| 23 | 0.263 | 0.821 | 93.627 | - | - | - | - | - | - |
| 24 | 0.257 | 0.804 | 94.431 | - | - | - | - | - | - |
| 25 | 0.257 | 0.802 | 95.233 | - | - | - | - | - | - |
| 26 | 0.244 | 0.762 | 95.995 | - | - | - | - | - | - |
| 27 | 0.237 | 0.740 | 96.735 | - | - | - | - | - | - |
| 28 | 0.229 | 0.716 | 97.451 | - | - | - | - | - | - |
| 29 | 0.219 | 0.685 | 98.136 | - | - | - | - | - | - |
| 30 | 0.204 | 0.637 | 98.773 | - | - | - | - | - | - |
| 31 | 0.200 | 0.624 | 99.397 | - | - | - | - | - | - |
| 32 | 0.193 | 0.603 | 100.000 | - | - | - | - | - | - |

*Supplementary Material 7. Rotated Factor Loadings of the Digital Competence Framework for Pre-service Physical Education Teachers (PSPETs) (n=719)*

| Item | Factor Loading | | | | | | | Communality |
| --- | --- | --- | --- | --- | --- | --- | --- | --- |
|  | Factor 1 | Factor 2 | Factor 3 | Factor 4 | Factor 5 | Factor 6 | Factor 7 |  |
| Q1 | 0.111 | 0.142 | 0.806 | 0.143 | 0.156 | 0.145 | 0.091 | 0.756 |
| Q2 | 0.127 | 0.173 | 0.820 | 0.166 | 0.081 | 0.155 | 0.086 | 0.784 |
| Q3 | 0.116 | 0.129 | 0.836 | 0.135 | 0.125 | 0.187 | 0.068 | 0.803 |
| Q4 | 0.139 | 0.237 | 0.780 | 0.190 | 0.078 | 0.186 | 0.084 | 0.767 |
| Q5 | 0.133 | 0.129 | 0.766 | 0.338 | 0.083 | 0.109 | 0.152 | 0.777 |
| Q6 | 0.327 | 0.772 | 0.155 | 0.100 | 0.148 | 0.148 | 0.042 | 0.783 |
| Q7 | 0.121 | 0.806 | 0.154 | 0.153 | 0.182 | 0.143 | 0.107 | 0.776 |
| Q8 | 0.132 | 0.801 | 0.216 | 0.138 | 0.152 | 0.082 | 0.077 | 0.761 |
| Q9 | 0.134 | 0.840 | 0.111 | 0.121 | 0.122 | 0.134 | 0.073 | 0.788 |
| Q10 | 0.096 | 0.825 | 0.151 | 0.098 | 0.173 | 0.164 | 0.045 | 0.782 |
| Q11 | 0.154 | 0.182 | 0.101 | 0.169 | 0.812 | 0.115 | 0.164 | 0.796 |
| Q12 | 0.096 | 0.136 | 0.133 | 0.152 | 0.812 | 0.155 | 0.179 | 0.783 |
| Q13 | 0.144 | 0.165 | 0.130 | 0.224 | 0.768 | 0.078 | 0.155 | 0.735 |
| Q14 | 0.128 | 0.274 | 0.113 | 0.144 | 0.791 | 0.104 | 0.165 | 0.789 |
| Q15 | 0.851 | 0.112 | 0.118 | 0.142 | 0.097 | 0.065 | 0.029 | 0.786 |
| Q16 | -0.833 | -0.146 | -0.088 | -0.152 | -0.065 | -0.036 | -0.059 | 0.755 |
| Q17 | 0.854 | 0.106 | 0.062 | 0.132 | 0.083 | 0.054 | 0.027 | 0.773 |
| Q18 | 0.785 | 0.120 | 0.152 | 0.110 | 0.153 | 0.127 | 0.207 | 0.749 |
| Q19 | 0.818 | 0.161 | 0.111 | 0.128 | 0.124 | 0.111 | 0.150 | 0.774 |
| Q20 | 0.839 | 0.104 | 0.090 | 0.060 | 0.054 | 0.081 | 0.069 | 0.740 |
| Q21 | 0.113 | 0.168 | 0.203 | 0.177 | 0.131 | 0.757 | 0.253 | 0.768 |
| Q22 | 0.084 | 0.120 | 0.149 | 0.156 | 0.126 | 0.852 | 0.090 | 0.818 |
| Q23 | 0.109 | 0.105 | 0.200 | 0.180 | 0.084 | 0.810 | 0.148 | 0.780 |
| Q24 | 0.137 | 0.317 | 0.215 | 0.154 | 0.137 | 0.749 | 0.056 | 0.773 |
| Q25 | 0.137 | 0.123 | 0.138 | 0.112 | 0.342 | 0.176 | 0.755 | 0.783 |
| Q26 | 0.120 | 0.072 | 0.157 | 0.256 | 0.183 | 0.143 | 0.758 | 0.738 |
| Q27 | 0.193 | 0.100 | 0.109 | 0.124 | 0.171 | 0.173 | 0.811 | 0.792 |
| Q28 | 0.160 | 0.122 | 0.193 | 0.805 | 0.096 | 0.170 | 0.073 | 0.770 |
| Q29 | 0.116 | 0.156 | 0.151 | 0.826 | 0.086 | 0.096 | 0.117 | 0.773 |
| Q30 | 0.158 | 0.117 | 0.205 | 0.776 | 0.122 | 0.131 | 0.156 | 0.739 |
| Q31 | 0.159 | 0.125 | 0.161 | 0.718 | 0.289 | 0.170 | 0.135 | 0.714 |
| Q32 | 0.161 | 0.108 | 0.219 | 0.767 | 0.218 | 0.152 | 0.079 | 0.751 |

*Supplementary Material 8. Confirmatory Factor Analysis Path Diagram for the Pre-service Physical Education Teachers’ Digital Competence (PSPET-DC) Framework*

*Supplementary Material 9. Stepwise Comparison of Measurement Invariance Models Across the Seven Demographic Variables*

| Group | Model | χ² | df | CFI | RMSEA | ΔCFI | ΔRMSEA |
| --- | --- | --- | --- | --- | --- | --- | --- |
| Gender | M1 Configural Invariance | 1379.29 | 886 | .988 | .019 | — | — |
|  | M2 Metric Invariance | 1400.44 | 911 | .988 | .019 | .0001 | −.0003 |
|  | M3 Scalar Invariance | 1424.93 | 936 | .988 | .018 | .0000 | −.0003 |
| Academic Stage | M1 Configural Invariance | 1379.43 | 886 | .988 | .019 | — | — |
|  | M2 Metric Invariance | 1409.35 | 911 | .988 | .019 | −.0001 | −.0002 |
|  | M3 Scalar Invariance | 1448.73 | 936 | .987 | .019 | −.0004 | .0000 |
| GPA Ranking | M1 Configural Invariance | 2022.10 | 1329 | .983 | .022 | — | — |
|  | M2 Metric Invariance | 2065.49 | 1379 | .983 | .022 | .0002 | −.0005 |
|  | M3 Scalar Invariance | 2129.05 | 1429 | .982 | .022 | −.0003 | −.0002 |
| Institutional Tier | M1 Configural Invariance | 1959.00 | 1329 | .984 | .021 | — | — |
|  | M2 Metric Invariance | 2036.74 | 1379 | .983 | .021 | −.0007 | .0001 |
|  | M3 Scalar Invariance | 2319.35 | 1429 | .977 | .024 | −.0060 | .0031 |
| City Tier | M1 Configural Invariance | 1871.37 | 1329 | .987 | .020 | — | — |
|  | M2 Metric Invariance | 1917.64 | 1379 | .987 | .019 | .0001 | −.0004 |
|  | M3 Scalar Invariance | 1958.94 | 1429 | .987 | .019 | .0002 | −.0005 |
| First-Generation College Students | M1 Configural Invariance | 1314.26 | 886 | .990 | .018 | — | — |
|  | M2 Metric Invariance | 1341.70 | 911 | .990 | .017 | −.0001 | −.0002 |
|  | M3 Scalar Invariance | 1400.74 | 936 | .989 | .018 | −.0008 | .0004 |
| Low-Income Family | M1 Configural Invariance | 1514.41 | 886 | .985 | .021 | — | — |
|  | M2 Metric Invariance | 1533.56 | 911 | .985 | .021 | .0001 | −.0004 |
|  | M3 Scalar Invariance | 1568.30 | 936 | .985 | .021 | −.0002 | −.0001 |

*Note: Measurement invariance was considered supported when ΔCFI ≥ −.01 and ΔRMSEA ≤ .015.*

*Supplementary Material 10. Comparison of the Dimensions of Four Mainstream General Frameworks with the Subject-Specific Framework for Physical Education Developed in This Study*

|  | This Study | DigCompEdu | UNESCO ICT-CFT V3 | ISTE Educator Standards | China’s Teachers’ Digital Literacy |
| --- | --- | --- | --- | --- | --- |
| Organizational Logic | 7 Main Categories × 25 Sub-Categories | 6 Functional Areas × 22 Competences | 3 Development Levels × 6 Aspects of Practice = 18 Competence Modules | 7 Teacher Roles | 5 First-Level Dimensions × 13 Second-Level Dimensions |
| Dimension 1 | A. Digital Resources for Physical Education Teaching | Digital Resources | Application of Digital Skills | Designer | Knowledge and Skills in Digital Technologies |
| Dimension 2 | B. Integration and Implementation in Physical Education Teaching | Teaching and Learning | Pedagogy | Facilitator | Digital Application |
| Dimension 3 | C. Assessment and Feedback in Physical Education Teaching | Assessment | Curriculum and Assessment | Analyst | Digital Application |
| Dimension 4 | D. Digital Ethics and Safety in Physical Education Teaching | — | — | Digital Citizen | Digital Social Responsibility |
| Dimension 5 | E. Professional Activities | Professional Engagement | Teacher Professional Learning | Learner/Collaborator | Professional Development |
| Dimension 6 | F. Fostering Students’ Use of Digital Technology for Health Maintenance | Empowering Learners’ Digital Competence | — | Facilitator | — |
| Dimension 7 | G. Personality Traits in Digital Technology Application | — | — | — | Digital Awareness |

*Note: The general frameworks differ fundamentally in their organizational logic. For example, the ISTE standards organize the framework around teacher roles rather than functional areas. To ensure systematic and replicable comparisons, this table aligns dimensions according to the principle of functional equivalence. Specifically, conceptual mappings were established based on the similarity of the functional content represented by the first-level dimensions of each framework. For example, the ISTE role of Analyst is functionally aligned with the assessment dimension of this study, whereas the role of Facilitator corresponds simultaneously to Integration and Implementation in Physical Education Teaching (B) and Fostering Students’ Use of Digital Technology for Health Maintenance (F).*
